# Research note: a novel framework for investigating chronic intestinal stressors in commercial broiler chickens

**DOI:** 10.1016/j.psj.2025.105864

**Published:** 2025-09-19

**Authors:** Christos Gougoulias, Alireza Khadem, Joan Torrent

**Affiliations:** a Innovad Group, Cogels Osylei 33 2600 Berchem, Belgium; b Lab of Nutrition, Faculty of Veterinary Medicine, Ghent University, Heidestraat 19 9820 Merelbeke, Belgium

**Keywords:** Chronic, ‘Low-grade’ intestinal inflammation, Model, Broilers

## Abstract

Traditional experimental models typically fail to capture the multifactorial stressors present in commercial environments, limiting their ecological validity. This study introduces a novel model designed to replicate cumulative production pressures by integrating mini-pens within an operational broiler house and applying bacterial enteritis (BE) scores and total mean lesion scores (TMLS) for *Eimeria spp.* as intestinal health indicators at fixed time points (days 21 and 28), where birds were subjected to a diet rich in non-starch polysaccharides (NSPs) without enzyme supplementation to induce chronic intestinal stress (CS). Performance metrics and intestinal health indicators (BE and TMLS scores) were compared across three environments: CS birds, a conventional production farm and a controlled research facility (the birds in the latter two were fed a standard corn/soybean diet). At day 35, birds in the CS setup were significantly lighter (1.999 kg) and less efficient (feed conversion:1.642) than the research facility birds, representing perhaps the maximum genetic potential (2.751 kg, feed conversion: 1.268). Bacterial enteritis scores at day 21 were highest in the CS group (3.75), compared to the Research Farm (2.25) and Production Farm (2.12), but in line with industry-reported dysbiosis levels. However, and interestingly, the relative reduction between day 21 and day 28 was similar only between the CS and the PF (∼12 %) but pronounced in the RF group (56 %), likely reflecting the absence of cumulative stressors under typical research conditions. Total microscopic lesion scores (TMLS) remained low overall, although they were slightly elevated in CS at both days 21 (1.38) and 28 (1.12), relative to the conventional Production Farm (0.63 and 0.38 at days 21 and 28, respectively). Histological analysis showed no significant differences in villus-to-crypt ratios between CS and the conventional production farm, suggesting physiological comparability. Thus, the CS model successfully captured realistic subclinical gut stress, bridging the gap between laboratory conditions and commercial practices. It provides an ecologically valid platform for evaluating nutritional and management interventions targeting gut health.

## Introduction

Modern broiler production is characterized by rapid growth rates aimed at achieving maximum body weight in the shortest possible time before slaughter. This accelerated growth is increasingly linked to metabolic syndromes, or production-related disorders, rather than traditional infectious diseases, particularly when birds are slaughtered after 40 days of age. These issues largely result from genetic selection favoring high early growth rates (Korver, 2023). The rapid growth has created a mismatch in the maturation of organs, with the gastrointestinal tract and its microbiota developing more slowly than the musculoskeletal system (Tallentire et al., 2016). Similarly, the immune system often remains incompletely developed up to slaughter, which can occur between 26 and 60 days of age depending on production systems (Song et al., 2021). Consequently, modern broilers are more susceptible to intestinal leakage and chronic gut inflammation (Pan and Yu, 2014).

A major limitation across most existing studies is the lack of ecological validity as the experimental conditions often do not realistically reflect commercial farming environments. To overcome these limitations, we propose a novel experimental framework that integrates *in situ* exposure to commercial production environments by installing experimental pens within a functioning broiler house, thereby subjecting the experimental birds to as many of the same environmental and managerial stressors as the ones experienced by the production flock. Unfortunately, certain stressors, such as those associated with animal density, cannot be accurately replicated, and therefore the resulting stress levels will inevitably be lower than those observed in commercial production farms. However, it is possible to introduce an additional stressor by providing a diet rich in non-starch polysaccharides (NSPs) without the inclusion of NSP-degrading enzymes, thereby intentionally inducing intestinal stress when baseline stress levels are considered insufficient. Finally, two macroscopic intestinal health related metric systems (‘bacterial enteritis’ and ‘total mean lesion scores’ due to *Eimeria* spp.) are performed at two specific time points of the bird’s life cycle. Therefore, the objectives of this study were to assess a chronic intestinal inflammation model by comparing it to research and commercial farm conditions.

## Materials and methods

The animal care and use procedures of the experiments were approved by the local ethics committee for animal experiments at Poulpharm Belgium (EC = C21331).

### Animals and facilities

Data were collected under 3 different experimental conditions:

1) Commercial Farm Birds (FB): Data were collected from a flock housed inside a commercial birdhouse accommodating approximately 55,000 birds (Ross 308). These birds were reared under standard commercial practices. All diet phases included coccidiostats (Table 1).

**Table 1:** Ingredient composition and calculated nutrient levels of the Commercial, Chronic Stress, and Research Facility diets (g/kg).

Ingredient	Commercial					Chronic Stress			Research Facility		
	Starter		Grower		Finisher	Starter	Grower	Finisher	Starter	Grower	Finisher
Corn	369.85		315.61		253.53	-	-	-	544.00	569.65	589.65
Wheat	200.00		320.00		418.00	613.35	592.25	653.80	-	-	-
Rye	-		-		-	-	50.00	-	-	-	-
Soybean meal 48	300.00		263.50		198.00	250.00	203.00	180.00	-	-	-
Soybean meal 46	-		-		-	-	-	-	382.00	350.00	315.46
Oats	25.00		21.25		12.00	-	-	-	-	-	-
Potato protein^1^	-		4.25		-	-	-	-	-	-	-
Rapeseed meal 33	-		-		-	40.00	45.00	50.00	-	-	-
Sunflower meal Hipro	-		-		36.00	20.00	30.00	-	-	-	-
Sunflower meal 27	-		-			-	-	25.00	-	-	-
Soybean oil	22.00		20.40		48.00	40.00	19.00	25.00	32.00	44.00	60.50
Peas	50.00		21.25		30.00	-	-	-	-	-	-
Palm fat	-		8.50		-	-	-	-	-	-	-
Animal lard	-		-		-	-	30.00	40.00	-	-	-
Vit-Min Premix^2^	5.00		4.25		4.98	5.00	5.00	5.00	5.00	5.00	5.00
Limestone	11.50		7.91		8.40	13.80	11.20	10.00	14.80	14.30	13.60
CaH_4_P_2_O_8_	6.00		3.74		-	8.20	3.00	2.00	8.80	7.50	6.90
NaCl	2.00		1.70		1.80	1.80	1.50	1.70	2.20	2.20	2.20
NaHCO_3_	2.00		1.70		2.40	1.70	2.90	1.20	2.50	2.50	2.20
L-Lysine HCl	2.60		2.38		2.40	2.60	3.35	2.95	2.85	1.90	1.95
DL-Methionine	3.10		2.76		1.92	2.50	2.40	2.05	3.95	2.20	2.05
L-Threonine	0.80		0.68		0.42	1.00	1.15	1.05	1.30	0.70	0.80
Valine	-		-		-	-	-	0.20	0.55	-	0.10
Phytase^3^	0.10		0.085		-	-	-	-	0.05	0.05	0.05
Xylanase^4^	0.05		0.043		-	-	-	-	-	-	-
Nutrient levels											
CP	212.8		201.0		184.1	215.7	200.0	189.6	225.3	209.7	195.1
ME, kcal/kg	3031		3123		3243	2933	3075	3113	3043	3140	3267
Lys	13.20		11.97		10.41	12.05	11.75	10.57	14.42	12.82	11.89
Met + Cys	9.61		9.00		7.74	9.03	8.74	7.97	10.83	8.76	8.20
Thr	8.48		7.83		6.73	8.33	7.87	7.32	9.70	8.60	8.12
Ca	8.1		6.0		5.9	9.00	7.30	6.50	8.50	8.00	7.50
Available P	4.5		3.6		3.1	4.50	3.60	3.00	4.90	4.60	4.40
Na	1.4		1.2		1.4	1.60	1.50	1.40	1.60	1.60	1.50

1 Protastar, Avebe, Prins Hendrikplein 20, 9641 GK Veendam, The Netherlands.

2 Vitamin-Mineral Premix (per kg premix): Vitamin A: 2,000,000 IU; Vitamin D_3_: 500,000 IU; Vitamin E: 10,000 mg; Vitamin K_3_: 460 mg; Vitamin B_1_: 400 mg; Vitamin B_2_: 1,500 mg; Vitamin B_6_: 700 mg; Vitamin B_12_: 4,000 μg; Niacin: 7,000 mg; d-pantothenic acid: 2,400 mg; Choline chloride: 92,000 mg; Folic acid: 200 mg; Biotin: 40 mg; Iron: 1,600 mg; Copper: 2,400 mg; Manganese: 17,000 mg; Zinc: 12,000 mg; Iodine: 240 mg; Selenium: 50 mg.

3 Axtra PHY (500FTU): Danisco Animal Nutrition, IFF, Willem Einthovenstraat 4, Oegstgeest 2342 BH, The Netherlands.

4 Ronozyme WX5000C: DSM Firmenich, Wilhelminasingel 39, 6221 BE, Maastricht, Netherlands.
2) Chronic stress (CS): This condition simulated the cumulative environmental conditions of a commercial broiler house. Eight floor metal pens were installed within the FB commercial broiler house. Each pen housed 30 birds, corresponding to a stocking density of 14 birds/m². Birds were subjected to a dietary challenge designed to induce chronic intestinal inflammation by replacing corn with NSP-rich cereals in the feed (Table 1).
3) Research Facility Birds (RB): This condition included 80 Ross 308 male broilers housed in a separate controlled research facility. Birds were allocated to eight mini-floor pens, with 10 birds per pen (stocking density: 6 birds/m²). Birds received a standard corn-soy-based diet without coccidiostats (Table 1).

Diets for the 3 conditions were formulated to meet nutrient requirements according to the genetic company standards (Ross 308 performance, standards). The diets were formulated to highlight the differences in intestinal stress levels between the ideal conditions of a research farm and those experienced under field conditions, as well as to assess whether the stress levels under in CS birds were comparable to those observed in FB birds.

### Growth parameters

Performance parameters were measured for CS and RF birds on days 14, 28, and 35. Feed intake was recorded daily, and feed conversion rate (FCR) values were corrected for mortality. For the PF birds, the final average BW, FI, and FCR were recorded at the conclusion of the production cycle (day 35).

### Sample collections

***Intestinal Scores.*** On days 21 and 28, 1 bird per pen from the CS and the RF groups and 8 visually healthy PF birds were randomly selected and euthanized by cervical dislocation. The intestinal tract of each bird was examined and evaluated for 2 macroscopical scores:

a) Total intestinal mucosa lesions (TMLS) to assess lesions indicative of *Eimeria* spp. infections (Johnson and Reid, 1970).
b) The bacterial enteritis (BE) score (Teirlynck et al., 2011) was used to evaluate signs of intestinal dysbiosis.

***Histomorphometry.*** At day 28, one bird per pen from both the CS and RF groups was randomly selected and euthanized by cervical dislocation. For histological analysis and measurements of villus height and crypt depth, tissue samples of the intestine (mid duodenum and mid jejunum) were taken (12 measurements per intestinal segment), immersed in formalin (1 %), fixed in Bouin’s solution for 24 h, and embedded in paraffin wax until use. Sections of 4 *μ*m were cut using a microtome (Microm; Prosan, Merelbeke, Belgium). After deparaffinisation, the sections were rehydrated in isopropylene (5 min), 95 % alcohol (v/v) (5 min), and 50 % alcohol (5 min) and stained with haematoxylin and eosin. Villus length and crypt depth in the jejunum were randomly measured in 3 villi per section (of all intestinal segments) using an Olympus BX61 Digital Camera DP50 (Olympus NV, Aartselaar, Belgium) and an image analysis system (Analysis w J-2; P4 Technologies, Inc., Waldorf, MD).

### Statistical analyses

For performance variables, a repeated measures design was employed. Prior to modeling, Levene’s test was conducted for each dependent variable to assess the homogeneity of variances across time points. When significant results indicated violations of the sphericity assumption, Linear Mixed Effects Models including fixed effects (day, treatment and their interaction) and random intercepts (to account for within-subject correlation) were fitted for each performance variable using the statsmodels package in Python (Python Software Foundation, version 3.11). Model fitting was performed using Restricted Maximum Likelihood. Interaction terms were included to assess whether treatment effects varied over time. For non-parametric variables (TMLS and BE), the Kruskal–Wallis test was applied to assess group differences, followed by Dunn’s test for post-hoc pairwise comparisons (R Core Team, 2013).

## Results and discussion

Performance differed significantly between the CS and RF treatments (Table 2). Birds reared in the RF environment achieved an average final BW of 2.751 kg and an FCR of 1.268, compared to 1.999 kg BW and an FCR of 1.642 in the CS system at day 35 (*P* < 0.001). In the PF setting, birds reached an average BW of 2.200 kg and an FCR of 1.534 (statistical comparisons were not possible as only production averages were available). However, direct comparisons between the PF and the other two groups, yielded, on one hand, a ∼550 g (25 %) increase in final BW and a ∼27-point improvement in FCR for the RF compared to the PF, and on the other hand a ∼760 g (38 %) increase in BW along with a ∼37-point improvement in FCR, when the RF averages were compared to the CS. It is worth noting that performance outcomes in the RF surpassed considerably the Ross 308 male performance standards at day 35 (BW: 2.334 kg; FCR: 1.443). Conversely, the CS system yielded ∼9 % (200 g) lower BW and an 11-point FCR reduction compared with the PF.

**Table 2:** Performance parameters for Chronic Stress and Research Farm birds.

		Period (days)				
BW gain		1-14	15-21	21-28	28-35	1-35
	Chronic Stress	0.504	0.349	0.568	0.578	1.999
	Research Farm	0.527	0.609	0.807	0.808	2.751
	SEM	0.006	0.008	0.007	0.018	0.025
	Power	0.74	1.00	1.00	1.00	1.00
	Treatment, *P*-value	<0.01	<0.01	<0.01	<0.01	<0.01
	Treatment × Day, *P*-value	0.13	<0.01	<0.01	<0.01	<0.01
Intake						
	Chronic Stress	0.550	1.004	0.879	1.242	3.283
	Research Farm	0.602	0.808	1.081	1.305	3.488
	SEM	0.006	0.008	0.018	0.025	0.038
	Power	1.00	1.00	1.00	1.00	0.94
	Treatment, *P*-value	<0.01	<0.01	<0.01	<0.01	<0.01
	Treatment × Day, *P*-value	<0.01	<0.01	<0.01	<0.01	<0.01
FCR^1^						
	Chronic Stress	1.091	1.683	1.549	2.157	1.642
	Research Farm	1.143	1.240	1.340	1.618	1.268
	SEM	0.004	0.015	0.029	0.041	0.009
	Power	1.00	1.00	1.00	1.00	1.00
	Treatment, *P*-value	<0.01	<0.01	<0.01	<0.01	<0.01
	Treatment × Day, *P*-value	<0.01	<0.01	<0.01	<0.01	<0.01

1 FCR: Feed Conversion Ratio (g:g).

Unexpectedly, the RF birds were less efficient (*P* < 0.01) at day 14 than the CS, with FCR values of 1.143 and 1.091, respectively. However, the difference in FCR would diminish if adjusted for body weight, as RF birds were proportionally heavier than the CS at day 14 despite the higher FCR.

At 21 days, BE scores were significantly higher in the CS group (3.75) compared to the RF (2.25) and PF (2.12) groups, which did not differ from each other. A similar pattern was observed at 28 days, with CS showing higher scores (1.38) than RF (0.00) and PF (0.63), suggesting that the inclusion of NSP exacerbated dysbiosis. However, and interestingly, the relative reduction between day 21 and day 28 was similar only between the CS and the PF (∼13 % and 12 %, respectively), but not so in the RF group (56 %). The reduced dysbiosis in the RF group likely results from the controlled, hygienic conditions of the experimental setting and the absence of cumulative commercial challenges. In contrast, the PF group exhibited substantially lower dysbiosis scores, performing well below the global industrial BE average and thus, representing a high-performing system. The CS group dysbiosis matches levels reported in broilers subjected to controlled gut leakage experiments that mimic cumulative production stress typical of modern farming (De Meyer et al., 2019).

Although the CS group exhibited numerically higher TMLS scores at both day 21 and day 28 (1.38 vs. 0.63 at day 21, and 1.13 vs. 0.38 at day 28, for the CS and PF groups, respectively), these differences were not statistically significant. Notably, no coccidial lesions were observed at any time point in the RF group, which highlights that research facilities may not mirror actual production conditions. Between days 21 and 28, TMLS scores fell by 40 % in the PF group and 18 % in the CS group. The latter prolonged mild lesions were likely due to the NSP challenge, which probably extended subclinical coccidiosis. The villus-to-crypt ratio at day 28 did not differ between the CS and the PF birds (∼7.0 and ∼5.0, for duodenal and jejunal ratios, respectively), showing that the CS model accurately mimicked what occurs under real production conditions. In summary, we present a novel approach using mini-pens within standard broiler production to study chronic intestinal inflammation and measure the gap between actual performance and genetic potential.

## CRediT authorship contribution statement

**Christos Gougoulias:** Supervision, Project administration. **Alireza Khadem:** Supervision, Investigation, Data curation. **Joan Torrent:** Writing – review & editing, Writing – original draft, Supervision.

## Disclosures

The authors declare that they have no conflict of interest related to this research

## Appendix Supplementary materials

Image, application 1
