# Supplementary material for: Research note: a novel framework for investigating chronic intestinal stressors in commercial broiler chickens
Source: Poult Sci. 2025 Sep 19;104(12):105864. doi: 10.1016/j.psj.2025.105864 (PMC12513191; doi:10.1016/j.psj.2025.105864)
Supplement: Supplementary file 1 [file mmc1.docx]

*AI statement*

During the preparation of this work the author(s) used Chat-GPT and Co-Pilot to improve the clarity of the text. After using this tool/service, the author(s) reviewed and edited the content as needed and take(s) full responsibility for the content of the publication
